# Bacteriome Diversity of Blackflies’ Gut and Association with *Onchocerca volvulus*, the Causative Agent of Onchocerciasis in Mbam Valley (Center Region, Cameroon)

**DOI:** 10.3390/pathogens11010044

**Published:** 2021-12-31

**Authors:** Arnauld Efon Ekangouo, Hugues C. Nana Djeunga, Guilhem Sempere, Joseph Kamgno, Flobert Njiokou, Paul Moundipa Fewou, Anne Geiger

**Affiliations:** 1 Centre for Research on Filariasis and other Tropical Diseases (CRFilMT), Yaoundé P.O. Box 5797, Cameroon; arnauld.efon@gmail.com (A.E.E.); kamgno@crfilmt.org (J.K.); anne.geiger@ird.fr (A.G.); 2 UMR InterTryp, IRD (Institut de Recherche Pour le Développement), University of Montpellier, F-34394 Montpellier, France; guilhem.sempere@cirad.fr; 3 Department of Biochemistry, Faculty of Sciences, University of Yaoundé I, Yaoundé, Cameroon; pmoundipa@hotmail.com; 4 South Green Bioinformatics Platform, Biodiversity, F-34934 Montpellier, France; 5 UMR InterTryp, CIRAD (Centre de Coopération Internationale en Recherche Agronomique Pour le Développement), Campus International de Baillarguet, F-34398 Montpellier, France; 6 Faculty of Medicine and Biomedical Sciences, University of Yaoundé I, Yaoundé, Cameroon; 7 Department of Animal Biology and Physiology, Faculty of Sciences, University of Yaoundé I, Yaoundé, Cameroon; njiokouf@yahoo.com

**Keywords:** onchocerciasis, blackfly, *Onchocerca volvulus*, bacteriome, next generation sequencing

## Abstract

Vector control using larvicides is the main alternative strategy to address limits of preventive chemotherapy using ivermectin for the control of onchocerciasis. However, it remains substantially limited by implementation difficulties, ecological concerns and the resistance of vector populations. Therefore, efficient and environmentally safe alternative control strategies are still needed. This study explores the composition of the blackfly bacteriome and its variability in the presence of *Onchocerca volvulus* infection, in order to determine their potential as a novel vector control-based approach to fight onchocerciasis. An entomological survey of a collection of samples was performed in the Bafia health district, a historical endemic focus for onchocerciasis in Cameroon. A total of 1270 blackflies were dissected and the infection rate was 10.1%, indicative of ongoing transmission of onchocerciasis in the surveyed communities. Sequencing process of blackflies’ gut DNA for bacteria screening revealed 14 phyla and 123 genera, highlighting the diversity of gut blackflies bacterial communities. Eight bacteria formed the core of blackfly bacteriome and *Wolbachia* was the predominant genus with 73.4% of relative abundance of blackflies’ gut bacterial communities. *Acidomonas* and *Roseanomas* genera were significantly abundant among infected blackflies (*p* = 0.01), whereas other genera such as *Brevibacterium* and *Fructobacillus* were associated with the absence of infection (*p* = 0.0009). Differences in gut bacterial distribution of blackflies according to their infection status by the parasite suggest a causal relationship between the bacteriome composition and the onset of blackflies’ infection by *O. volvulus* or vice versa. Blackfly native bacteria are then potentially involved in infection by *O. volvulus*, either by facilitating or preventing the parasite infestation of the vector. These bacteria represent an interesting potential as a biological tool/target for a novel approach of vector control to fight onchocerciasis.

## 1. Introduction

Onchocerciasis or river blindness is an infectious disease caused by the parasitic nematode, *Onchocerca volvulus.* The vectors are blackflies belonging to the genus *Simulium*; they are arthropods that breed in the oxygenated waters of fast-flowing rivers [1]. Following ingestion of microfilariae by the vector during its blood meal, the first stage larva penetrates the midgut wall and migrates to the fly muscles where it molts twice. The third stage larva migrates to the head of the blackfly [1,2] and penetrates the skin of human, the only known natural vertebrate host of *O. volvulus*, during a subsequent blood meal. The larvae migrate to the subcutaneous tissue where they form nodules and undergo successive molt until the adult stage, with an average lifespan estimated at 10–15 years [2,3]. After maturation and mating, adult females will release, for their entire life, 200,000–400,000 microfilariae per three-monthly reproductive cycle. Microfilariae may then invade the dermis causing skin conditions, as well as eye tissues causing various eye lesions (keratitis, iridocyclitis, etc.) which ultimately result in permanent blindness [4]. Indeed, onchocerciasis is the second leading cause of blindness from infectious origin after trachoma in Africa [5].

The Global Burden of Disease Study estimated in 2017 that there were 20.9 million prevalent *O. volvulus* infections worldwide: 14.6 million of the infected people had skin disease and 1.15 million had vision loss [6]. Africa has the highest burden of the disease, with 99% of the infection and 1.49 million disability-adjusted life years (DALYs) annually. Onchocerciasis has been reported to be significantly associated with epilepsy [7,8] and excess mortality [9,10] among people living in endemic areas.

Ivermectin was registered for the control of onchocerciasis in 1987 [11,12]. Since its main effect on *O. volvulus* is microfilaricidal, and adult worms are lifelong, treatments should be repeated annually or multi-annually to expect transmission interruption. Preventive chemotherapies through the community-directed treatment with ivermectin (CDTI) strategy led to the interruption of the transmission of the disease in four of the six onchocerciasis foci in Latin America [13,14]. However, despite almost three decades of preventive chemotherapy in Africa, onchocerciasis remains a public health problem in many countries, including Cameroon [15,16]. Indeed, recent epidemiological surveys carried out between 2011 and 2015, revealed the persistence of onchocerciasis with microfilarial prevalence higher than 60% in certain foci in the Centre, Littoral and West Regions despite more than two decades of CDTI [15,16]. The reasons related to this situation appear to be multifactorial, including (i) high proportion of permanent non-compliers infected persons living in endemic areas [17,18] (ii) foci located in conflict and hard to reach zones [12], (iii) sub-optimal responses of *O. volvulus* to ivermectin [19,20], and (iv) very high transmission levels due to high densities of blackflies with important vector competence [21]. These factors constitute tremendous obstacles to the process of elimination of the disease [22,23].

In order to accelerate the interruption of the transmission process, various complements or alternatives to the classical CDTI approach, so-called alternative/complementary treatment strategies, have been considered, including vector control [24,25]. However, the classical vector control approach based on the weekly use of larvicides, either aerial or ground larviciding in blackflies infested breeding sites, remains limited by the implementation difficulties, the significant risks of ecological pollution and fairly substantial implementation costs [26] and foci specificities constraints related to the geography and the size of rivers which are substantially important [17,27]. Additionally, re-colonization of blackflies after treatment of breeding sites has been observed in some foci.

These vector control difficulties being shared by other vector-borne diseases, mitigation or alternative approaches are likely to be the same. Previous studies in other vectors (tsetse and mosquitoes) demonstrated the impact of their microbiome in the vector competence, as well as their promising role as effective tools/targets for new generations of vector control strategies [28,29,30]. It is now well known that certain native bacteria species such as *Wigglesworthia glossinidia*, an obligate intracellular bacteria of tsetse intestinal cells, are necessary for the survival of their host [31,32]. While some bacteria are associated with the refractory character of their hosts to parasitic infection, others are associated with the reduction of the viability of their hosts in case of the parasite infestation. This evidence is observed with *Serratia mascesens,* which produces a trypanolytic compound preventing the establishment of *Trypanosoma cruzi* in the digestive tract of *Rhodnius prolixus* [31,33], while other *Serratia species* are associated with the reduction in *Anopheles* infection by *Plasmodium* [34].

Hence, native bacteria of vectors can be targeted and/or manipulated in different ways for vector control, notably as chemotherapeutic target, immunological reinforcement or cytoplasmic incompatibility, by inducing through genetic manipulation, a disturbance of molecular interactions between the parasite and vector host [29,35]. The prerequisite for the development of such a vector control approach is the identification and the characterization of native bacterial communities of the targeted vector and the assessment of their potential as effective tool/target for vector control. Thus, the discovery of such bacteria in blackflies would constitute a major breakthrough and will open wide avenues for the development of an innovative approach in the fight against onchocerciasis in Africa through the development of non-infestable blackflies. Hence, this study was designed to screen the whole bacterial communities of blackfly gut and highlight bacterial species associated with vector competence and those associated with vector refractoriness to *O. volvulus* infection and thus assess their possible impact on onchocerciasis transmission.

## 2. Results

### 2.1. Blackflies Infection by Onchocerca volvulus

From the 1270 dissected blackflies, a total of 207 (16.3%*;* 95% CI: 14.3–18.4) were parous and screened for *Onchocerca volvulus* infection (Table 1). Overall, 21 (10.1%; 95% CI: 6.7–15.0) blackfly guts were infected with *O. volvulus*. The infection rate was site dependent. Biatsota displayed the highest infection rate (13.0%; 95% CI: 7.8–20.1), followed by Bayomen (9.1%; 95% CI: 3.1–12.3) and Nyamongo (6.8%; 95% CI: 2.9–14.8), although the difference was not statistically significant (*p* = 0.405). Analysis of DNA samples from blackfly heads revealed five samples positive to *O. volvulus*, with an infectivity rate of 2.4% (95% CI: 1.0–5.5), and no significant differences in infectious blackflies distribution among geographic areas (*p* = 0.42).

### 2.2. Sequencing Data Analysis

**Figure 1:**
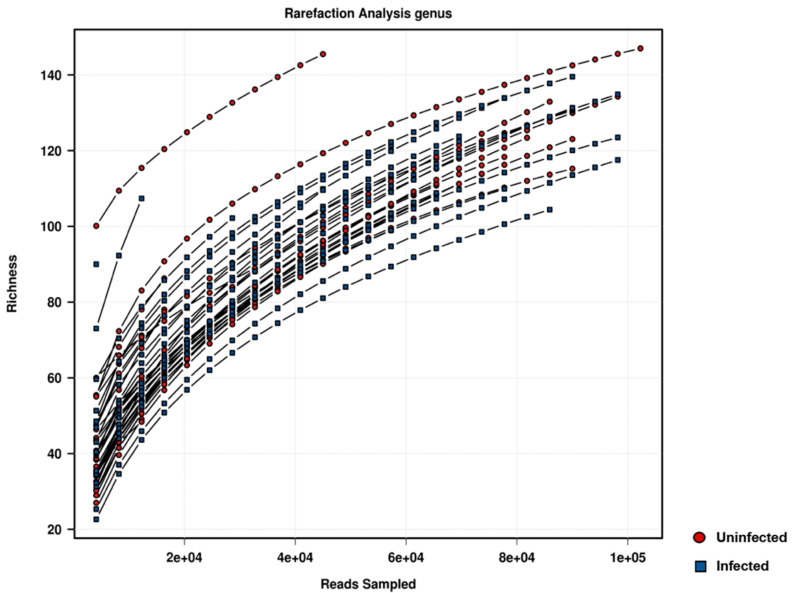
Rarefaction analysis on the blackfly samples. The red circles represent uninfected flies and the blue squares represent infected flies.

DNA from 42 parous blackflies (21 infected and 21 randomly selected among uninfected samples), were selected for sequencing. Sequencing of 16S rDNA from total DNA extracted from blackflies intestine using Illumina sequencing technology generated a total of 3,427,049 high quality sequence reads across the V3–V4 region. After filtering of contaminants, the average number of tags per sample for the V3–V4 region was 60,819 (ranging from 4832 to 102,633 per sample), with read length varying from 300 to 400 nucleotides. A rarefaction curve showed the saturation of most of the samples between 80,000 and 100,000 reads. This range corresponds to a minimum richness in Operational Taxonomic Units (OTUs) estimated at 100 for all samples (Figure 1).

### 2.3. Taxonomic Assignment

The taxonomic assignment of OTUs sequences allowed for the identification of 23 phyla, among which 22 belonged to the Bacteria kingdom and only one belonged to Archaea (*Euryarchaeota phylum*). For further analysis, rare taxa were filtered by excluding those presenting with less than 0.05% of relative abundance across all samples and those present in less than 10% of sequenced samples to exclude potential residual contaminants. Using these filters, a total of 14 phyla were found fulfilling defined criteria and retained for further investigations. Among retained phyla, *Proteobacteria* represented the most predominant bacteria phylum with 77.1% of global relative abundance across the 42 samples and represented more than 90% in proportion for 55% of samples. Other phyla were unevenly distributed among the different samples.

### 2.4. Bacterial Genera

#### 2.4.1. Global Distribution

**Figure 2:**
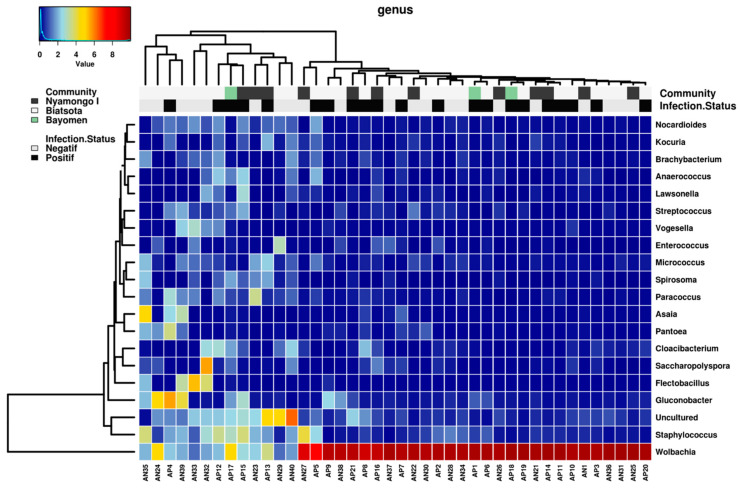
Heat map analysis of the distribution and abundance of the bacterial genera in blackfly gut samples. Samples are clustered from left to right: cluster 1 including samples AN35 to AN40 and cluster 2 including samples AN27 to AP20. The cluster 1 is divided into two sub-cluster: Sub-cluster 1A including samples from AN35 to AN39 and sub-cluster 1B including samples from AN33 to AN40.

A total of 554 bacterial genera was observed, with relative abundance ranging from 6.6E-05% to 70.2%. Likewise, prior to phyla analysis, successive filters were applied: exclusion of DNA extraction related contaminants, exclusion of bacterial genera with relative abundance lower than 0.05%, and those present in less than 10% of samples to exclude potential residual contaminants. After application of filters, a total of 123 genera were retained for further analyses. A heat map analysis showed that the hierarchical clustering of the bacterial relative abundance of 20 predominant bacterial genera across the 42 samples resulted in a poorly structured tree (Figure 2). Only *Wolbachia* with 73.4% of relative abundance (ranging from 1.46% (AN29) to 94.1% (AP19), respectively, as minimum and maximum relative abundance across all samples) was distinctly separated from the other genera. However, the map showed a slight contrast on these bacterial distribution with a more uniform color print at the right half of the heat map. This observation was strengthened by the hierarchical clustering of the samples on the basis of the relative abundance of considered bacterial genera, which allowed for discriminating between the two main clusters: cluster 1 including samples AN40 to AN35, and cluster 2 including AN27 to AP20 (Figure 2). However, the samples of either cluster 1 or cluster 2 seemed to be associated neither with infection status nor with geographic origin of sample. The hierarchical clustering of bacteria genera, based on blackflies and infection status and geographic area where they were captured, showed no specific bacterial clustering. However, *Wolbachia* showed a relative homogeneity of abundance distribution from samples AN27 to AP20.

#### 2.4.2. Blackfly Gut Core Bacteriome

**Figure 3:**
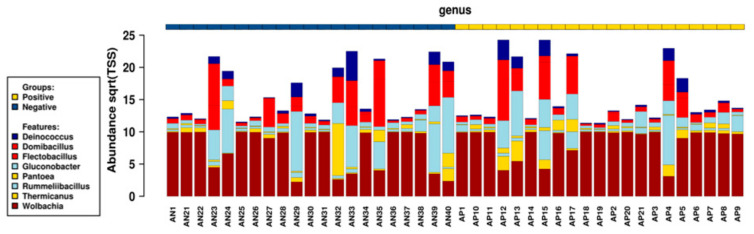
Normalized clustered group chart highlighting the profiles of core bacterial genera for individual samples. The height of colored bar indicates the relative abundance of bacterial genus associated with corresponding sample. Samples are organized regarding the infection status from AN1 to AN40 for uninfected blackflies and from AP1 to AP9 for infected blackflies.

Eight bacterial species were systematically present in all samples (Figure 3). These bacterial genera with global relative abundance estimated at 89.3% considering the overall taxa, could possibly represent the core of blackfly gut bacteriome. Wolbachia was the most represented bacterial genus (*p* < 0.0001) with relative abundance of 84.4% of the core bacteriome, followed by Gluconobacter and Flectobacillus genera, with relative abundance of 2.2% and 1.7% among OTUs, respectively. The less abundant bacterial genera were Deinococcus and Thermicanus, with a relative abundance of 0.3% and 0.2%, respectively.

### 2.5. Association between Blackflies’ Bacterial Diversity with Infection Status

**Figure 4:**
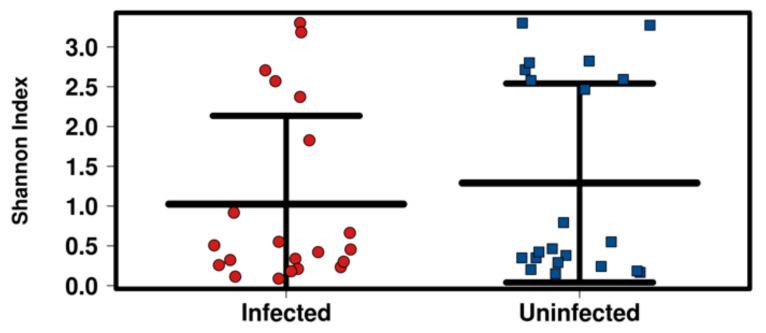
Alpha diversity using Shannon Index assessing the relationship between the bacterial diversity of blackflies’ gut with blackfly infection status (infected vs. non-infected).

The potential relationship between the bacterial diversity of blackflies’ gut, with either the infection status or geographical origin, was investigated by estimating the α-diversity using the Shannon Index, which measures overall diversity, including both the number of OTUs and their evenness. No significant differences were observed either in bacterial diversity and richness regarding the infection status (infected vs. non-infected) of sampled blackflies (*p* = 0.349) (Figure 4).

**Figure 5:**
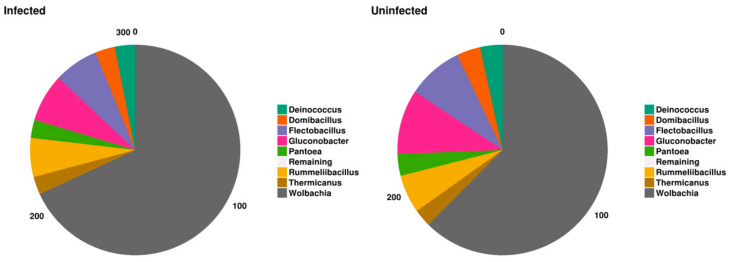
Normalized pie chart showing the composition of blackflies core bacteriome regarding the status of infection.

Investigating the potential factors associated with the variability of the core bacteriome components (Figure 5) revealed that despite the increasing relative abundance of *Wolbachia* in the presence of parasite (infected blackflies) (*p* = 0.2), the proportion of the majority of the core bacteria genera was globally similar regarding the infection status of blackflies. Considering the overall bacterial composition of blackflies’ gut (including bacteriome core components), the presence of parasite (infected blackflies) was negatively associated with the bacteriome variability.

### 2.6. Multivariate Association between Bacterial Diversity in Blackflies and O. volvulus Infection Status

**Figure 6:**
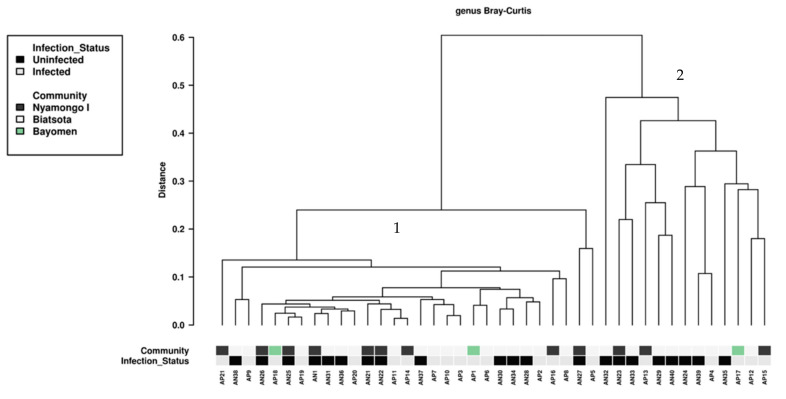
Bray–Curtis multivariate analysis of complex association between bacterial composition and explanatory variables (community of origin and infection status) across the 42 selected samples. Two main clusters are highlighted here: cluster 1 I from AP21 to AP5 and cluster 2 from AN32 to AP15.

The investigation of potential relationships between the gut bacterial communities of blackflies and their infection status (infected vs. uninfected) was done to highlight potential complex associations between gut blackfly bacterial composition and the other covariate. A hierarchical clustering using the Bray–Curtis index did not discriminate unambiguously the different groups regarding the infection status (Figure 6). However, it showed a structure quite similar (symmetrical from the point of view of the layout) to the one on heat map (Figure 3), with two main clusters, cluster 1 from A21 to AP5 and cluster 2 from AN32 to AP15.

### 2.7. Potential Biomarker According to Blackfly Infection Status

In order to determine potential biomarker(s) with reference to bacterial genera associated with specific feature (infected/uninfected status) of sampled parous blackflies, we performed richness analysis of each taxonomic unit in different conditions using feature selection method (Figure S1). Table 2 below summarizes the most significant biomarkers associated with infection status. Several bacterial genera were significantly associated with infected status including *Acidomonas*, *Roseamomas*, *Cnuella* and *Serratia*, whereas other bacterial genera were significantly associated with uninfected status, including *Fructobacillus, Sanguibacter, Phaseolibacter* and *Brevibacterium*. At the level of species, *Unclassified Wolbachia* was significantly associated with infected blackflies whereas *Unclassified Micrococcus*, *Fructobacillus Unclassified Fructobacillus* were significantly abundant in uninfected blackflies.

## 3. Discussion

This study’s aim to characterize the whole bacterial community within the blackflies’ gut and to assess their potential associations with vector competence is, to our knowledge, pioneering in onchocerciasis. Nonetheless, this approach based the on successful outcomes of parallel studies on other vector-borne diseases is a fundamental prerequisite for application of vector control strategy-based on modified non-infestable blackflies to gradually reduce disease transmission in onchocerciasis endemic areas.

The global infestation rate was 10.0% in the Bafia health district, suggesting that onchocerciasis transmission is still ongoing in this historically endemic area, despite almost three decades of community-directed treatment with ivermectin (CDTI). Our results confirm previous evidences supportive of ongoing transmission both within the human population (microfilarial prevalence ranging from 24.4 to 57.0%) [36] and in vector population (>98% of infection detected in blackflies using pool screening approach) [37]. The level of endemicity in the surveyed communities of the Bafia health district contrasts with the therapeutic coverage of the annual round of mass drug administration, which increased from 35% in 2001 to reach 80% in 2013 in the Centre Region, showing a better coverage of the targeted population [36]. This epidemiological situation might be related to the proximity of the surveyed communities to the Mbam River, as shown on the map of the study area. This river is characterized by a series of rapids and well-oxygenated water that provide ideal breeding sites for blackflies’ development. These observations are supportive of the importance of vector control approach in the process of elimination of onchocerciasis [24,38].

According to both infestation and infectivity rates, Biatsota was considered as the most active community in terms of disease transmission; this situation is likely to be related to its geographical situation which is closer to the Mbam River as compared to other surveyed communities. Importantly, in this community, economic activities are more intense along the river, thus increasing the biting rate in contrast to Bayomen and Nyamongo communities where inhabitants are living less close to the river, hence contact with blackflies are less frequent.

In this study, taxonomic assignment allowed us to identify a total of 14 phyla and 123 genera. This result highlights the diversity of gut blackflies bacterial communities, which seems significantly larger than the bacterial diversity reported in many recent studies on other arthropods of medical importance such as tsetse and mosquitoes. Indeed, studies on tsetse, using the same molecular approach, showed that the gut bacteria communities were made up of 14 phyla and 83 different bacteria genera [31,32]; meanwhile, sequencing of 16S rRNA sequences in *Aedes aegypti, Ae. albopictus*, and *Culex quinquefasciatus* using 453 pyrosequencing technique revealed that they were made up of less than six bacterial phyla [39].

Our findings revealed that Proteobacteria was the predominant phylum of the core bacteriome of blackfly. This phylum common to several insects [31,32,35,40], plays a major role in energy and nutrient metabolism and is involved in activities such as fatty acid and sugar metabolism, degradation of organic compounds, and biosynthesis of vitamin cofactors such as vitamin E (tocopherols) [41,42]. *Wolbachia* was the most important bacterial genus of blackflies’ gut bacteriome. This result is in agreement with previous estimates suggesting that *Wolbachia* infects more than 65% of all insect species [43], though they are also widespread and common in other invertebrates such as arachnids and crustaceans [44,45]. Beyond their presence and their likely role in vector biology, *Wolbachia* is also an important endosymbiont of numerous worms of medical importance such as the main filarial parasites (*Onchocerca volvulus, Brugia malayi, Mansonella perstans* and *Wuchereria bancrofti*) [44,46,47] except *Loa loa* [48,49] where it is known to play a substantial role in their development and pathogenesis in human hosts.

The analysis of bacterial taxa from blackflies’ gut did not show significant differences in bacterial composition on blackflies originating from the three surveyed communities. This could be explained by the fact that the three selected communities, Bayomen, Biatsota and Nyamongo, are located in the same geographical area (Bafia health district), within ~20 km and share both the same bio-ecological (climate, flora) and environmental features. These observations were similar to those recorded on *Anopheles* [50] where no significant differences of the bacterial flora were found between mosquitoes collected in similar ecological features foci in Cameroon. Such evidence was also observed with tsetse [31,32], demonstrating that bacterial composition of flies collected in Campo and Bipindi, two foci sharing similar ecological features, were not significantly different. In the line of these studies, vector populations from distinct geographical area with different eco-climatic features are expected to share significantly different bacterial communities. Such a possibility was evidenced by Askoy et al. [51] who reported differences in bacterial composition between distinct populations of Glossina transmitting *Trypanosoma rhodesiense*. However, differences could not be exclusively associated with the ecological differences of surveyed foci, but also with tsetse species (*G. fuscipes fuscipes, G. morsitans morsitans* and *G. pallidipes*) that are commonly found in different biotic and abiotic habitats. In this frame, even though *Simulium damnosum* complex is known as the important vector for *O. volvulus* in Cameroon [37,52,53], *S. yahense* and *S. squamosum* are associated with forest and forest–savannah transitional zones [37,52]. Further studies should be conducted on *Simulium* genetics of these localities to ensure if the highlighted homogeneity of bacterial communities within captured blackflies is shared by a common *Simulium* species or if there are different *Simulium* species with similar bacterial communities.

The investigation of bacterial genera (biomarkers) specifically associated with the infection status of blackflies showed a significant association between the abundance of some genera (*Serratia, Acidomonas, Roseamomas* and *Cnuella*) and the presence of the parasite. These bacteria potentially improve the susceptibility of blackflies to *Onchocerca volvulus* infection. Among these biomarkers, only *Serratia. sp* has been described in other arthropod vectors and its role seems to be vector-dependent. In Mosquitoes, *Serratia odorifera* has been associated with the susceptibility of *Aedes aegypti* both to chikungunya virus [54] and dengue virus [55]. Meanwhile, other studies demonstrated the ability of *Serratia marcescens* to produce some trypanolytic compounds that increase the refractoriness of *Rhodnius prolixus* to *T. cruzi* infection [33]. *Serratia* has also been described as facultative bacteria in blackflies [56]. However, further investigations are needed to decipher the host–bacteria interactions as well as to assess whether the biological role is mediated by single bacteria species or by the whole significantly associated bacteria genera. This evidence illustrates the complexity of molecular interaction with biological impact on vector susceptibility or refractoriness to parasite infection according to the bacterial species. Besides, other bacteria genera, in particular *Brevibacterium*, were found to be significantly associated with the absence of infection among blackflies. This Gram-negative bacterium was not yet reported to play a biological role in any vector-borne disease, thus opening a potential research avenue with possible outcome of interest. Beside genera, *Wolbachia Unclassified Wolbachia* was the single bacteria species significantly associated with the presence of the parasite infection in blackflies. *Wolbachia* are commonly classified in different super groups showing an asymmetric distribution within a large host range including both arthropod and filarial hosts. Further studies need to be undertaken to fully describe *Wolbachia Unclassified Wolbachia*. However, the overwhelming presence of the genus *Wolbachia* as a result of the sequencing of the whole blackflies’ gut bacteriome could have lowered the efficiency of amplification process of low abundant or rare bacteria genera with potential biological implications, hence increasing the sequencing depth associated with exclusion of *Wolbachia* amplification could increase the probability of detecting other rare taxa with potential biological role of interest for vector control.

In addition to these questions about the possible association between intestinal bacteria and the susceptibility/resistance of blackflies to infection with *O. volvulus*, a supplementary question is arising from the structure of the hierarchical clustering shown in Figure 2 and Figure 6. The 42 samples are clearly distributed into two clusters that neither the geographic origin nor the infection status can explain. Considering all these samples are coming from blackflies belonging to the *Simulium damnosum* complex, one cannot incriminate a possible differentiation related to a species difference. The simple observation of the heat map highlights contrast in colors intensity which represents differences in abundance of the various bacteria and allows us to discriminate the two clusters. Hence, existence within the vector population, of a genetic diversity (existence of different genotypes) could be at the origin of the observed structuration. Thus, it appears necessary to explore, besides the possible involvement of intestinal bacteria in blackflies infection, such a hypothesis in further investigation in order to get a better insight into the complex interactions between the three partners—the blackfly, its intestinal bacteria and the parasite, which are together responsible for the transmission of onchocerciasis.

## 4. Limitations

The main limitation of this study is the fact that the methodology did not allow us to determine the precise anatomic location of identified bacterial in the vector since the whole abdomen of blackfly was considered for sequencing. In addition, the number of uninfected blackflies since the bacteriome comparison did not take into account nulliparous blackflies to reinforce the postulate about the origin of some bacteria of interest (whether they are native to blackfly or originate from blood meals). Nevertheless, the methodology (control of sources of contamination during sampling/samples processing and consideration of negative controls) and the data analysis (use of high filtering thresholds) were sufficiently rigorous to detect and exclude potential contaminants an improve integrity of the data generated.

## 5. Materials and Methods

### 5.1. Ethics Approval and Consent to Participate

Although this study did not directly involve human subjects, samples (capture of blackflies) were collected using the human landing collection technique, which requires volunteers. Hence, an ethical clearance was obtained from the Centre Regional Ethics Committee for Human Health Research (N°1011/CRERSH/C/2020) and administrative authorizations were granted by the Centre Regional Delegate for Public Health and the Bafia District Medical Officer. Prior to the beginning of the entomological survey, the objectives and schedules of the study were explained to all the volunteers. Participation was entirely voluntary and each of them (aged 24 years and above) was free to opt out without fear of retaliation. The volunteers recruited lived in sampling sites, so they were not more exposed to fly bites than usual. Moreover, volunteers were trained to capture flies before being bitten. Finally, ivermectin was provided as preventive chemotherapy against onchocerciasis.

### 5.2. Study Area

This study was carried out in the Bafia health district, situated in Mbam and Inoubou Division, Centre Region, Cameroon. This health district is known for its historical endemicity to onchocerciasis and disease persistence despite two decades of ivermectin-based preventive chemotherapy. Communities of this health district are mainly watered by the Mbam River and its tributaries, whose falls and rapids promote and maintain blackfly breeding sites throughout the year. The phytogeography of this area shows a forest/savannah transition zone dominated by a peri-forest savannah with forest galleries along the rivers and important breeding sites favorable to the development of blackflies. Bafia is mainly dominated by the subequatorial climate with average temperature of 23.5 °C and bimodal rainfall regime marked by modest precipitations with average rainfall of 831.7 mm. Socio-economic activities are dominated by sand extraction in the Mbam river, as well as agriculture and trade on the shores of the latter.

### 5.3. Capture, Dissection and Preservation of Blackflies

**Figure 7:**
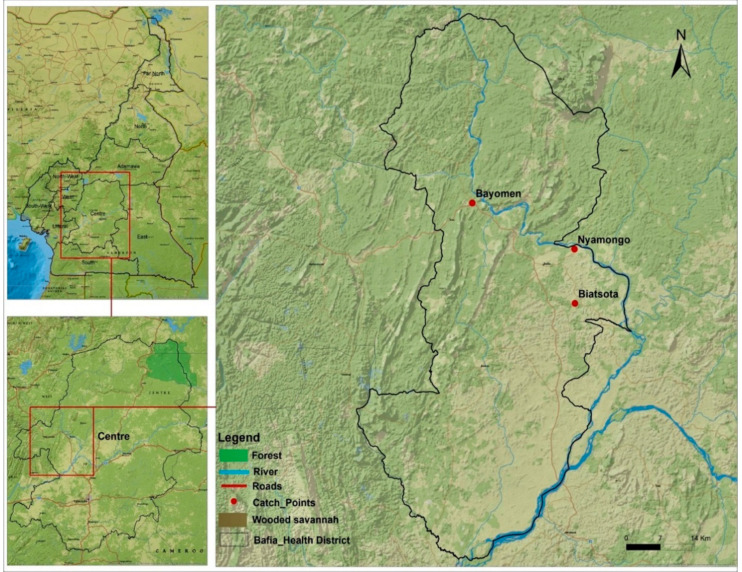
Map showing the locations of study sites.

Entomological surveys were conducted in April 2019 in three communities of the Bafia health district, namely Bayomen (04°51′52″ N; 11°06′07″ E), Biatsota (04°41′11″ N; 11°17′28″ E) and Nyamongo (04°46′57″ N; 11°17′24″ E) located between 5 and 10 km from the Mbam River (Figure 7). In each selected site, blackflies were captured using the “human Landing collection” method. Catches were made by two groups of volunteers, the first working from 8 a.m. until 1 p.m. and the second from 1 p.m. until 5 p.m. Adult female blackflies, which are hematophagous, landed on the exposed legs of well-trained community volunteers and captured before having time to take their blood meal.

From sample collection to statistical analysis of meta-taxonomic data, several measures have been taken into consideration to mitigate potential contaminant DNA and cross-contamination at each step of the process. Blackflies were collected using sterile tubes. Prior to the dissection process, captured blackflies were immersed for about 5 min in a saline solution supplemented with sodium hypochlorite (detergent) and individually isolated for dissection into a sterile drop of water. The dissection process was carried out in a wind-protected area to mitigate potential environmental contaminants and dissecting tools were systematically decontaminated after the dissection of each fly. Blackflies were described based on morphological criteria to ensure their belonging to *Simulium damnosum* complex [57,58] and primarily dissected for parity evaluation using a binocular stereo-microscope. A secondary dissection was performed on parous blackfly. Indeed, parous flies were dissected to investigate the presence of *O. volvulus* infection since they had already had at least one blood meal from potentially infected individuals, unlike nulliparous flies which are immature flies (never having experienced a blood meal) and, therefore, could not be infected by the parasite. This latter group of flies was not considered for further analysis. Dissected parous blackflies were separated into four parts: gut, thorax, head and legs, which were individually transferred into well labelled 1.5 mL An Eppendorf tube containing 70° Ethanol was stored at −20 °C for further molecular analysis.

### 5.4. DNA Extraction and O. volvulus PCR Amplification

Genomic DNA was extracted from heads and guts samples and purified on MiniElute PCR purification columns using the QIAamp DNA Mini kit (Qiagen Inc., Les Ulis, France) and eluted in 50 µL molecular biology-grade water. DNA extraction processes were carried out in an isolated environment (laminar-flow hood), surfaces and equipment were decontaminated and exposed to UV light for 15 min prior experiments.

DNA extracted from gut and head samples were processed for the detection of *O. volvulus* by quantitative PCR (qPCR) using specific primers (Forward: 5′-GCTATTGGTAGGGGTTTGCAT-3′ and reverse: 5′-CCACGATAATCCTGTTGACCA-3′) targeting a DNA portion (128 bp) of ND5 *O. volvulus* gene and probe (5′-FAM-TAAGAGGTTAAGATGG NFQ-3′). Each well of the microtiter plate (MicroAmp fast optical 96-well reaction plate, Applied Biosystems) was filled with 20 µL of final solution, containing 2 µL DNA template and 18 µL of PCR master mix made up of: 12 µL molecular biology-grade water, 2 µL of 10 × PCR buffer, 2.4 µL 50 × MgCl2 (50 mm), 0.1 µL dNTPs (10 mm), 0.6 µL forward primer (10 mm), 0.6 µL reverse primer (10 mm), 0.2 µL ND5 *O. volvulus* probe, and 0.1 µL HotStarq polymerase (5 U/µL). For each amplification process, negative and positive controls were used to ensure good interpretation of final results. Real-time PCR assays were performed on an Applied Biosystems Step One Plus real-time PCR machine (Applied Biosystems, Foster City, CA, USA) using the following cycling conditions: initial denaturation at 95 °C for 10 min, followed by 45 cycles, each including a denaturation step at 95 °C for 1 min, an annealing and elongation step at 60.1 °C for 30 s.

### 5.5. High Throughput Sequencing and Meta-Barcoding Analysis

The 16S rRNA gene V3-V4 variable region was amplified using specific primers designed in the scope of a previous study [33] to assess the bacterial communities of blackfly guts using the Illumina MiSeq sequencing approach (MR DNA Laboratory, http://www.mrdnalab.com/shallowater (accessed on 21 December 2021), Shallowater, Texas, USA). PCR was performed using the HotStar Taq Plus Master Mix Kit (Qiagen Inc., Texas, USA) under the following conditions: 94 °C for 3 min for initial denaturation, followed by 30 cycles of successive steps: denaturation at 94 °C for 30 s, annealing at 53 °C for 40 s and elongation at 72 °C for 1 min, and a final elongation step at 72 °C for 5 min. After amplification, PCR products were checked on 2% agarose gel to determine the success of amplification and the relative intensity of bands. Multiple PCR products were pooled together in same proportions based on their molecular weight and DNA concentrations. Pooled PCR products were purified using calibrated Ampure XP beads (Details on Manufacturer). Then, the pooled and purified PCR product were used to prepare Illumina DNA library. Sequencing process was performed at MR DNA (www.mrdnalab.com) (accessed on 21 December 2021), Shallowater, TX, USA) using a MiSeq following the manufacturer’s guidelines.

PCR blanks (where DNA free water was used instead of DNA template) were run as a control process for generated libraries. If PCR blanks showed any signal (likely contaminants), the experiment was repeated, as the PCR control (blank PCR) under 30–35 cycles for a library should not generate any signal.

Prior to running the metabarcoding pipeline, a specific reference file for the assignment step was generated. This was achieved by running CutAdapt v1.8 (http://dx.doi.org/10.14806/ej.17.1.200) (accessed on 21 December 2021) with those primers to extract V3-V4 reference sequences from the SILVA SSU database (release 132). The generated sequences were deposited in the EMBL-EBI (study accession number: PRJEB38276; secondary study accession number: ERP121684).

The first stage in the workflow consisted of filtering read quality using CutAdapt with a threshold value of 20. Then, VSearch v2.14 (https://dx.doi.org/10.7717%2Fpeerj.2584) (accessed on 21 December 2021) was used in combination with CutAdapt for the following series of tasks: (i) merging the forward and reverse reads of each sample; (ii) demultiplexing to obtain one fastq file per sample; (iii) clipping barcodes and primers; (iv) excluding sequences containing unknown bases; (v) calculating expected error rate; and (vi) performing sample-level dereplication. The remaining sequences were then pooled into a single FASTA file to allow VSearch to carry out a global dereplication, after which clustering was applied to remaining sequences using Swarm v2.2.2 (https://dx.doi.org/10.7717%2Fpeerj.1420) (accessed on 21 December 2021). VSearch was then used again to identify chimeric clusters.

The STAMPA (https://github.com/frederic-mahe/stampa) (accessed on 21 December 2021) pipeline was then run for taxonomic assignment of representative OTU sequences based on the contents of the specific reference file generated from SILVA SSU records. This generated an OTU table to which the following filters were applied in order to retain targeted taxa at genus level: elimination of clusters with a high expected error (>0.0002) and elimination of small clusters (less than 3 sequences) observed in a single sample.

### 5.6. Statistical Analysis

Data obtained from bioinformatics analysis of raw data (Table S1) was formatted in QIIME OTU table and analyzed with metadata sheet using Calypso 8.84 (https://dx.doi.org/10.1093%2Fbioinformatics%2Fbtw725) (accessed on 21 December 2021), an online software dedicated to bacterial taxonomic analysis.

Infection and infectivity rates were computed as the proportion of parous blackflies harboring *O. volvulus* parasites at the level of gut and head, respectively. Parity rate was calculated as the proportion of dissected blackflies that had already taken a blood meal at least once (parous blackflies). For all proportions, the exact (Clopper–Pearson) 95% confidence intervals (95% CI) were calculated as a dispersion parameter. Bacterial Relative richness was computed as the proportion of normalized operational taxonomic unit (OTU) among the overall OTUs identified, considering single, part or complete sequenced blackflies samples. Prior to data analysis, pre-filtering steps were performed to improve statistical analysis and counteracts sequencing errors. Filtering step included the removal of samples with less than 1000 sequence reads, removal of taxa having less than 0.05% of relative abundance across all samples. In addition, OTUs identified as contaminants (mainly associated with DNA extraction process) were filtered and excluded from the data set prior to data analysis. Total Sum Scaling (TSS) normalization combined with square root transformation (Hellinger transformation) was used to normalize count data by dividing feature read counts by the total number of reads in each sample. This method converted raw feature counts to relative abundance.

Heat map was used for intuitive visualization of the bacterial genera distribution among samples. It highlighted the contrast in bacterial composition with associated clustering trends and relationships between relative richness and sample distribution. Color histogram and camembert plots were used to summarize the blackflies’ gut core bacteriome composition, according to geographical origin and the infection status of blackflies. A dendrogram was used to represent the random clustering of sample based on bacterial composition in order to highlight potential association with co-variables (geographical origin or infection status of samples).

A Chi-squared test was used to assess the difference of the prevalence of infection between geographical origins. Given that OTU associated relative abundance are typically over dispersed and therefore do not follow a normal distribution, the non-parametric Mann–Whitney U and Kruskal–Wallis tests were used to compared average bacterial abundance between infection status and geographical origin of samples, respectively. Shannon and Simpson Indexes dedicated to the measurement of bacteria community heterogeneity were used to compare the diversity of different groups of samples (geographical origin and infection status) using raw data (not transformed or normalized by TSS). Shannon index was used to describe the disorder and uncertainty of individual genera; the uncertainty was proportional to the diversity, i.e., the diversity increased with the number of genera. Simpson diversity index was computed as the probability that two bacteria randomly sampled belong to different genera. The greater the Simpson index, the higher the diversity. For all analyses, the threshold of significance was set at 0.05.

## 6. Conclusions

This study exploring the blackfly bacteriome is, to our knowledge, pioneering in onchocerciasis. It reveals the existence of a core bacteriome of blackfly dominated by the genus *Wolbachia*. Some bacteria genera are significantly associated with the presence of *O. volvulus* in blackflies while others are refractory to it, giving an insight of biomarkers with interesting potential as biological tool/target for developing of non-infestable blackflies.

## Figures and Tables

**Table 1:** Parity and infection rates of collected blackflies according to geographic origin.

Geographic Origin	No. Dissected Flies	No. Parous Flies (Prop; 95% CI)	No. Parous Flies with *O. volvulus* (Prop; 95% CI)
Bayomen	200	34 (17.0%; 12.1–23.0)	3 (8.9%; 1.9–23.7)
Biatsota	558	100 (18.0%; 14.9–21.4)	13 (13.0%; 7.1–21.2)
Nyamongo	512	73 (14.3%; 11.3–17.6)	5 (6.9%; 2.3–15.3)
Total	1270	207 (16.3%; 14.3–18.4)	21 (10.1%; 6.4–15.1)

No.: number of; prop: proportion; CI: Confidence Interval.

**Table 2:** Bacterial genera identified as biomarkers with significant association to infections.

Bacterial Genera	*p*	AUC (Lower–Upper)	Odds Ratio Infected/Uninfected	Mean Infected	Mean Uninfected
*Fructobacillus*	0.0009	0.79 (0.65–0.93)	0.45	0.06	0.18
*Roseomonas*	0.01	0.73 (0.57–0.89)	1.41	0.36	0.17
*Acidomonas*	0.01	0.72 (0.56–0.87)	7.12	0.09	0.07
*Phaseolibacter*	0.014	0.72 (056–0.88)	2.97	0.07	0.09
*Sanguibacter*	0.016	0.71 (0.55–0.87)	3.80	0.05	0.08
*Jatrophihabitans*	0.017	0.68 (0.54–0.07)	0.07	0.01	0.07
*Leuconostoc*	0.019	0.66 (0.54–0.79)	0.26	0.01	0.07
*HdN1*	0.021	0.67 (0.54–0.81)	0.26	0.01	0.06
*Brevibacterium*	0.028	0.70 0.54–0.86)	0.27	0.09	0.35
*Tanticharoenia*	0.033	0.69 (0.53–0.85)	2.24	0.08	0.10
*Cnuella*	0.04	0.35 (0.22–0.49)	6.25	0.08	0.02

## Data Availability

The datasets supporting the conclusions of this article are included within the article and its additional files. Sequencing data related to Simulium damnosum bacteriome, generated from this study were deposited in the EMBL-EBI (Study accession number: PRJEB38276; secondary study accession number: ERP121684).

## Supplementary Materials

The following are available online at https://www.mdpi.com/article/10.3390/pathogens11010044/s1, Figure S1: Forest plot illustrating the odd ratio variation of top 100 bacterial genera (candidate biomarkers) relative abundance depending on blackflies infection status (uninfected/infected), Table S1: Summary of the overall sequencing raw data regarding the 42 blackfly samples.
