# Supplementary figures and images for: Bacteriome Diversity of Blackflies’ Gut and Association with Onchocerca volvulus, the Causative Agent of Onchocerciasis in Mbam Valley (Center Region, Cameroon)
Source: Pathogens. 2021 Dec 31;11(1):44. doi: 10.3390/pathogens11010044 (PMC8779297; doi:10.3390/pathogens11010044)

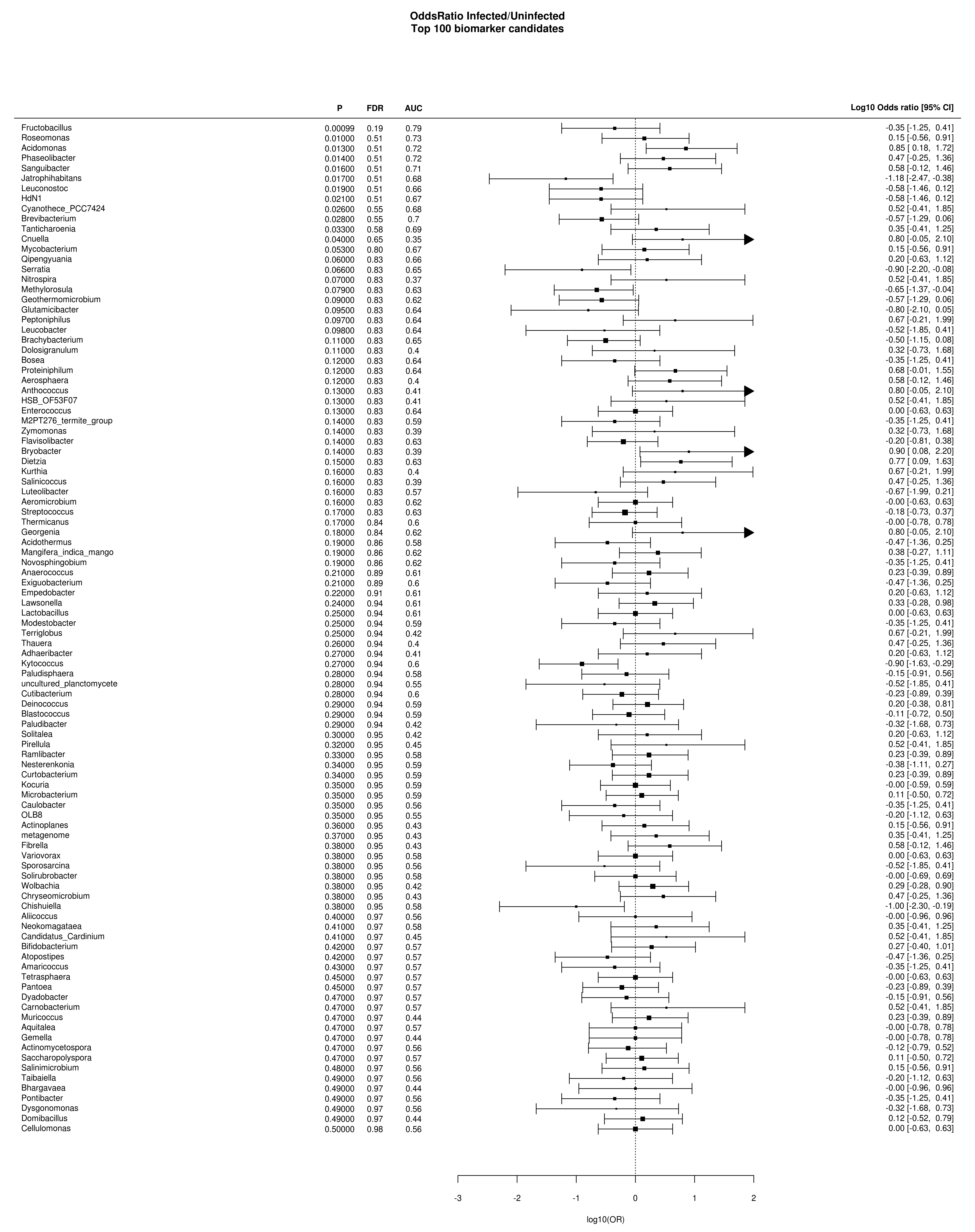

Supplement: Supplementary file 1 [file pathogens-11-00044-s001.zip › Figure S1.png]
